# A Horizontal-Gate Monolayer MoS_2_ Transistor Based on Image Force Barrier Reduction

**DOI:** 10.3390/nano9091245

**Published:** 2019-09-02

**Authors:** Kun Yang, Hongxia Liu, Shulong Wang, Wei Li, Tao Han

**Affiliations:** Key Laboratory for Wide-Band Gap Semiconductor Materials and Devices of Education, The School of Microelectronics, Xidian University, Xi’an 710071, China

**Keywords:** horizontal gate, MoS_2_, transistor, image force, barrier reduction

## Abstract

Transition metal dichalcogenides (TMDCs) have received wide attention as a new generation of semiconductor materials. However, there are still many problems to be solved, such as low carrier mobility, contact characteristics between metal and two-dimensional materials, and complicated fabrication processes. In order to overcome these problems, a large amount of research has been carried out so that the performance of the device has been greatly improved. However, most of these studies are based on complicated fabrication processes which are not conducive to the improvement of integration. In view of this problem, a horizontal-gate monolayer MoS_2_ transistor based on image force barrier reduction is proposed, in which the gate is in the same plane as the source and drain and comparable to back-gated transistors on-off ratios up to 1 × 10^4^ have been obtained. Subsequently, by combining the Y-Function method (YFM) and the proposed diode equivalent model, it is verified that Schottky barrier height reduction is the main reason giving rise to the observed source-drain current variations. The proposed structure of the device not only provides a new idea for the high integration of two-dimensional devices, but also provides some help for the study of contact characteristics between two-dimensional materials and metals.

## 1. Introduction

Since the discovery of graphene, two-dimensional materials have received extensive attention because of various peculiar physical phenomena. However, graphene with zero band gap is difficult to turn off when used in transistors [1], has a very low switching ratio and, therefore, is not suitable for digital integrated circuits [2]. Interestingly, transition metal dichalcogenides (TMDs) have shown potential advantages in electrical devices, with MoS_2_ becoming the research hotspot in recent years. Compared with graphene, MoS_2_ has a wide band gap, so that field-effect transistors with on-off ratios up to 10^9^ have been obtained [3]. At the same time, the energy band structure depends on the thickness of MoS_2_. Monolayer MoS_2_ is a direct band gap semiconductor with band gap of about 1.8 eV, while multilayer MoS_2_ exhibits indirect band gaps with widths ranging between 1.2 and 1.7 eV, which means MoS_2_ has good photoelectric characteristics [4,5,6].

However, the widespread application of MoS_2_ is limited by some undesired factors, such as low carrier mobility. To address this limitation, a great deal of research was carried out. It has been found that the performance of MoS_2_ transistors is limited by contact resistances, which are due to Schottky barriers [7]. In order to reduce the Schottky barrier height between MoS_2_ and metal, various methods have been used to improve the contact performance which includes selecting metals with a suitable work function and inserting a buffer layer such as graphene or boron nitride [8,9,10]. To promote the commercialization of MoS_2_ transistors, the complexity of the manufacturing process needs to be reduced [11,12,13]. It is worth noting that MoS_2_ is a layered material without dangling [14,15]. The resulting weak van der Waals interlayer interaction makes it difficult to deposit the dielectric on MoS_2_. Thus far, most of the research on MoS_2_ transistors is based on back-gate devices, which are not conducive to integration.

In this work, a monolayer MoS_2_ FET with a horizontal-gate structure is proposed making use of the principle of electrostatic induction and electrical characteristics comparable to back-gate transistors can be obtained. The table comparing the device with the literature is shown in Table S1 (the Supplementary Materials). In order to further understand its operation mechanism, it is verified that the mirror force barrier reduction is the dominant factor by combining the YFM method and the diode equivalent model.

## 2. Materials and Methods

**Figure 1:**
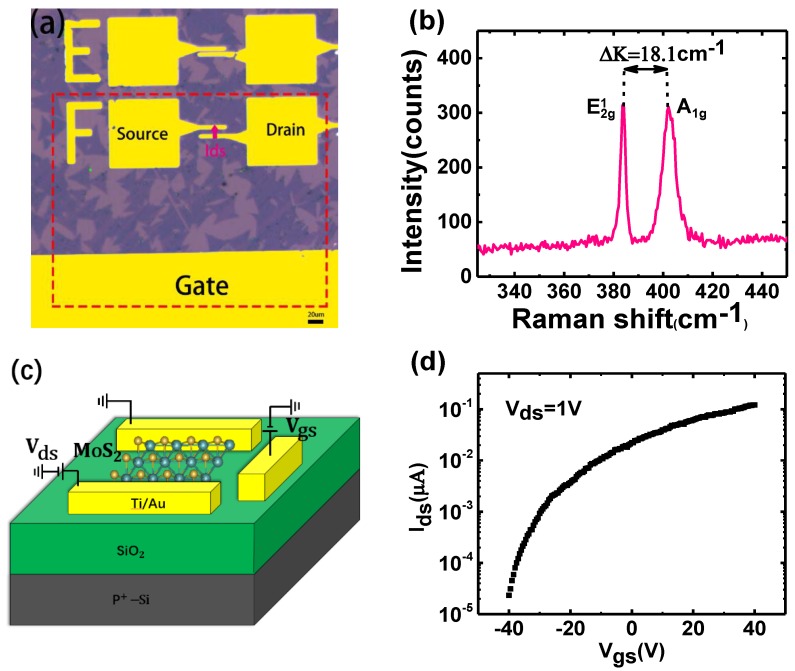
(**a**) The optical image of horizontal-gate device. (**b**) The Raman spectrum of monolayer MoS_2_ (**c**) The schematic of the horizontal-gate monolayer MoS_2_ transistor. (**d**) Transfer characteristic curve when Vds is equal to 1 V.

Monolayer MoS_2_ was synthesized on a P^+^Si/SiO_2_ substrate by chemical vapor deposition (CVD) [16,17] and the SEM images of monolayer MoS_2_ are provided in Figure S1 (Supplementary Materials). Gate/source/drain electrodes were defined by ultraviolet lithography. Then, Ti (20 nm)/Au (100 nm) electrodes were deposited by electron beam evaporation followed by resist removal. Subsequently, the device was annealed at 200 °C for 2 h under Ar_2_ atmosphere to remove water molecules adsorbed on the MoS_2_ [18]. Figure 1a shows an optical image of the horizontal-gate device, including all electrodes on the same plane. The thickness of MoS_2_ synthesized by CVD was characterized by Raman spectroscopy. As can be seen from Figure 1b, the wavenumber difference between the two peak positions is 18.1 cm^−1^, which is the typical characteristic of monolayer MoS_2_ [19]. Electrical measurements were performed with an Agilent B1500A analyzer (Santa Clara, CA, USA). All of the above measurements were performed at room temperature.

## 3. Results and Discussion

The transfer characteristic of the horizontal-gate monolayer MoS_2_ transistor is shown in Figure 1d, from which the on-off ratio up to 1 × 10^4^ can be obtained when the gate voltage is from −40 V to 40 V. Figure 1c shows the schematic of the horizontal-gate monolayer MoS_2_ transistor including all electrodes in the same plane, which can greatly improve the integration compared to the back gate MoS_2_ transistors.

**Figure 2:**
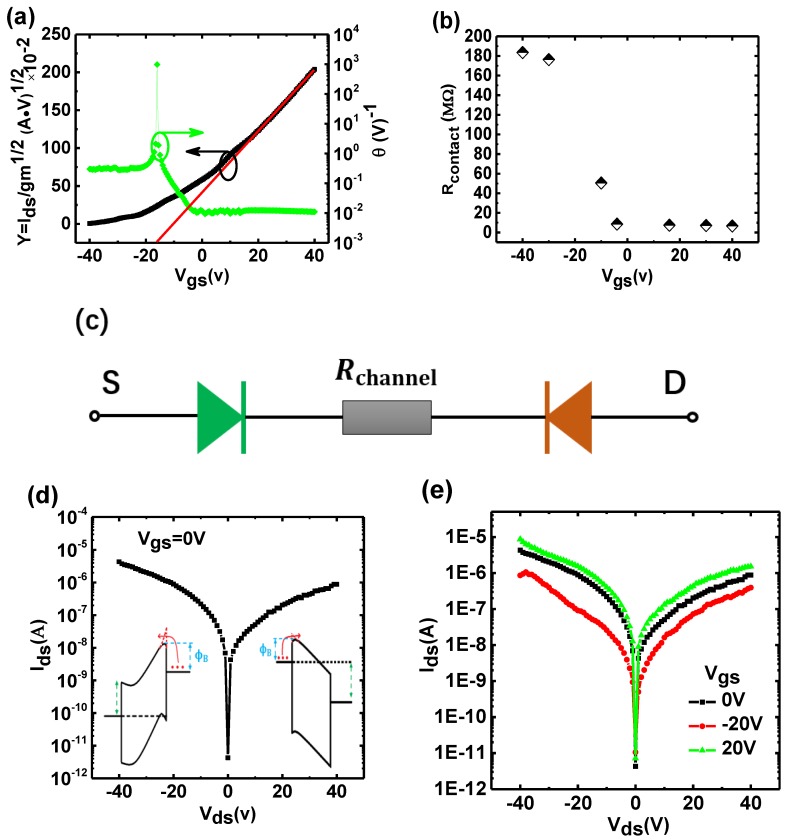
(**a**)The relationship curve between the mobility degradation coefficient θ and V_gs_ (green) and Y-function as function of V_gs_ (black). (**b**) The contact resistance extracted from the mobility degradation curve. (**c**) The equivalent diode model of current flowing. (**d**) I–V characteristics at V_gs_ = 0 V and the energy band diagram(inset). (**e**) I–V characteristics at different gate voltage.

In order to better understand the operation mechanism and the contact characteristics of the horizontal-gate monolayer MoS_2_ transistor, we need to extract important parameters including mobility, threshold voltage and contact resistance. By comparing the results of two-probe and four-probe measurements, it is proven that the YFM method can be used to effectively extract device parameters. All parameters are provided in Table S2 (Supplementary Materials). According to the YFM method, the Y-function is defined as [20]:

(1) $$Y=\frac{I_{ds}}{g_{m}{}^{1/2}}={\left(\frac{W}{L}C_{ox}V_{ds}\mu_{0}\right)}^{1/2}(V_{gs}-V_{th})$$

where $I_{ds}$ the drain current, $V_{gs}$ the applied horizontal-gate voltage,$\;g_{m}$ the trans-conductance, $C_{ox}$ the gate capacitance per unit area, $V_{th}$ the threshold voltage of the horizontal-gate transistor, L and W are the channel length and width, respectively. As shown in Figure 2a, we plot the Y-function as a function of gate voltage and linearly fit the linear region, from which the mobility and threshold voltage can be extracted from the slope and the intercept, respectively. The mobility degradation coefficient $\theta_{0}$ (Equation (3)) can be obtained from the modified current equation (Equation (2)). The relationship between the mobility degradation coefficient θ and the gate voltage is shown in Figure 2a. As the gate voltage increases, the mobility degradation coefficient tends to be stabilize when the horizontal-gate MoS_2_ transistor is operated in the linear region. To extract the contact resistance, the relationship between the mobility degradation coefficient and the contact resistance is required, that is, Equation (4):

(2) $$I_{ds}=\frac{WC_{ox}}{L}\cdot \frac{\mu_{0}}{\left[1+\theta (V_{gs}-V_{th})\right]}\left(V_{gs}-V_{th}\right)V_{ds}$$

(3) $$\theta =[(g_{m}(V_{gs}-V_{th})/I_{ds})-1]/\left(V_{gs}-V_{th}\right)$$

(4) $$\theta =\theta_{0}+R_{contact}C_{ox}\mu_{0}W/L$$

where $\theta_{0}$ is the intrinsic degradation coefficient of the mobility, which is so small that it can be ignored under normal conditions, only considering the effect on contact resistance at higher bias conditions. From the relationship θ with $V_{gs}$ shown in Figure 2a, the contact resistance at different gate voltages can be extracted to be about 6.7 KΩ, as shown in Figure 2b. Due to the existence of a Schottky barrier between the MoS_2_ and the metals electrodes, the contact resistance affected by the height of the Schottky barriers, the contact resistance decreases as the gate voltage increases. The contact resistance does not change uniformly with the gate voltage because the contact resistance is not only affected by the height of the Schottky barriers, but also by other factors such as the equivalent resistance due to the tunneling current induced by barrier width thinning, interlayer resistance and so on. According to two probe measurements [21], the channel resistance can be obtained from:

(5) $$R_{\mathrm{total}}=R_{\mathrm{channel}}+R_{\mathrm{contact}}$$

After obtaining the channel resistance of the device, the diode characteristics of the device can be measured, as shown in Figure 2e, in which a unique phenomenon was observed that the reverse current was greater than the forward current. The reason for this asymmetry is likely due to the different heights of the image barrier reduction on both sides as shown in the inset of Figure 2d. In order to verify this hypothesis, we explored the operation mechanism of the horizontal-gate transistor from the perspective of the diode model, shown in Figure 2c.

Considering the existence of a Schottky barriers at the source and the drain contacts, the current flow process can be seen as passing through two Schottky diodes connected back-to-back. Consequently one of the two diodes is always reverse biased, no matter how the voltage is applied. The main voltage drop occurs at the reverse biased diode and the channel resistance and can be evaluated according to the Equation (6).

**Figure 3:**
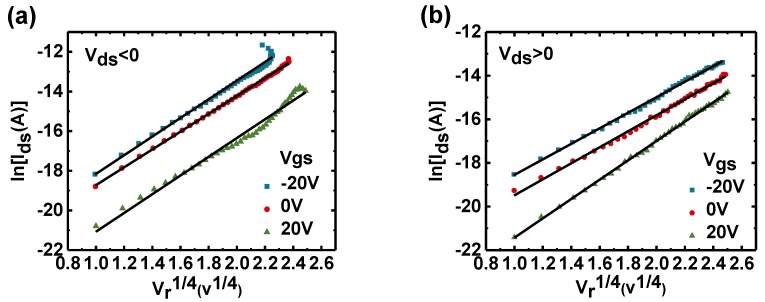
The linear relationship between $\ln I_{ds}$ and $\sqrt[{4}]{\left|V_{r}\right|}$. (**a**) Reverse direction (V_ds_ > 0). (**b**) Forward direction (V_ds_ > 0).

**Figure 4:**
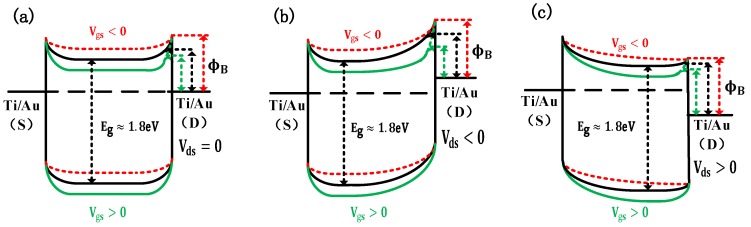
The energy band diagram at different gate voltage where V_gs_ < 0 (red), V_gs_ = 0 (black), V_gs_> 0 (green). (**a**) V_ds_ = 0 (zero bias). (**b**) V_ds_ < 0 (reverse bias). (**c**) V_ds_ > 0 (positive bias). We find it that both V_gs_ and V_ds_ have an effort on the Schottky barrier heights. The Schottky barrier heights reduction is the most obvious when V_gs_ > 0 and V_ds_ < 0 (figure b green line).

The typical trait of the Schottky barrier reduction due to the image force is that the logarithm of the current depends on the fourth root of the bias voltage [22,23]. Figure 3 shows the function relationship between ln (I_ds_) and $\sqrt[{4}]{\left|V_{r}\right|}$, from which the similar linear relation was obtained. This not only proves the correctness of the diode model, but also indicates that the Schottky barrier reduction induced by image force is the reason of current increasing. The result is consistent with the following expression [24]:

(6) $$V_{r}=V_{\mathrm{ds}}-R_{\mathrm{channel}}I_{\mathrm{ds}}$$

(7) $$I_{\mathrm{ds}}=AA^{*}T^{2}\exp\left[\frac{q}{kT}\left(\alpha \sqrt[{4}]{\left|V_{r}\right|}-\phi_{B}\right)\right]$$

where A is the area of the junction, A* is the Richardson’s coefficient, α is the dimensional constant, and $\phi_{B}$ is the Schottky barrier height. It can be seen from the above results that the characteristics of the diodes under different gate voltages are consistent with the change trend caused by the Schottky barrier height reduction subjected to image force. However, from the perspective of the applied bias voltage, the diode characteristics at different gate bias voltages appear to be transistor characteristics controlled by the gate and drain voltages. Hence, we have reason to believe that the operation mechanism of the horizontal-gate transistor is related to the Schottky barrier reduction caused by the image force, which can be explained from the angle of the energy band model of Figure 4, which shows the variation of the energy bands for different gate voltages and different drain voltages. It is worth noting that the polarity and magnitude of the gate and drain voltages all affect the amount of the image charge, inducing the difference in reduced barrier height. The difference of the image force dependence on gate voltage resulted in the variation of Schottky barrier height, which caused the on-state current increasing or decreasing. In contrast to a previous report [25], which believed that the variation of tunneling current caused by gate voltage is the main cause of on-state current increasing. We find that the reduction of Schottky barrier heights due to image force effects is the main reason for current increase. This discrepancy is mainly because the gate-control capability of the proposed horizontal-gate transistor is not sufficient to generate tunneling due to a wider Schottky barrier, so that the reduction of the Schottky barrier height caused by the image force becomes the dominant factor. It is important to understand the tunneling and Schottky barrier height reduction how to affect the operation of device for improving the performance of device, because the contact between metal and MoS_2_ is a key factor affecting device performance [3,26,27].

## 4. Conclusions

In this work, a horizontal-gate MoS_2_ transistor based on image force barrier reduction is proposed. The contact resistance of the device was extracted by the YFM method, and the dependence of the electrical characteristics of the device on the image force was verified by the diode model. Finally, the operation mechanism of the horizontal-gate transistor was explained from the perspective of the energy band models. Compared with back-gate transistors, horizontal-gate transistor not only has comparable electrical characteristics, but also feature simplified fabrication processes that do not require deposition of the dielectric layers, which facilitates integration. At the same time, this work also indicates that the interface characteristics are not only affected by the tunneling effect, but also influenced by the mirror force barrier reduction, which promotes the study of interface characteristics to a certain extent.

## Supplementary Materials

The following are available online at https://www.mdpi.com/2079-4991/9/9/1245/s1, Figure S1: The SEM images of monolayer MoS_2_ and transistor, Table S1: Comparison of the performance of device with literature. Table S2: Typical values of parameters in equations.

## References

[1] Radisavljevic B., Radenovic A., Brivio J., Giacometti V., Kis A. (2011). Single-layer MoS_2_ transistors. Nat. Nanotechnol..

[2] Liu W., Kang J., Sarkar D., Khatami Y., Jena D., Banerjee K. (2013). Role of metal contacts in designing high-performance monolayer n-type WSe_2_ field effect transistors. Nano Lett..

[3] Illarionov Y.Y., Smithe K.K., Waltl M., Knobloch T., Pop E., Grasser T. (2017). Improved hysteresis and reliability of MoS_2_ transistors with high-quality CVD growth and Al_2_O_3_ encapsulation. IEEE Electron. Device Lett..

[4] Di Bartolomeo A., Pelella A., Liu X., Miao F., Passacantando M., Giubileo F., Grillo A., Iemmo L., Urban F., Liang S.J. (2019). Pressure-Tunable Ambipolar Conduction and Hysteresis in Thin Palladium Diselenide Field Effect Transistors. arXiv.

[5] Lin J., Li H., Zhang H., Chen W. (2013). Plasmonic enhancement of photocurrent in MoS_2_ field-effect-transistor. Applied. Phys. Lett..

[6] Kim C., Moon I., Lee D., Choi M.S., Ahmed F., Nam S., Cho Y., Shin H.J., Park S., Yoo W.J. (2017). Fermi level pinning at electrical metal contacts of monolayer molybdenum dichalcogenides. ACS Nano.

[7] Islam A., Lee J., Feng P.X.L. (2018). All-dry transferred single-and few-layer MoS_2_ field effect transistor with enhanced performance by thermal annealing. J. Appl. Phys..

[8] Wang J., Yao Q., Huang C.W., Zou X., Liao L., Chen S., Fan Z., Zhang K., Wu W., Xiao X. (2016). High mobility MoS_2_ transistor with low schottky barrier contact by using atomic thick h-BN as a tunneling layer. Adv. Mater..

[9] Kim G.S., Kim S.H., Park J., Han K.H., Kim J., Yu H.Y. (2018). Schottky Barrier Height Engineering for Electrical Contacts of Multilayered MoS_2_ Transistors with Reduction of Metal-Induced Gap States. ACS Nano.

[10] Kaushik N., Karmakar D., Nipane A., Karande S., Lodha S. (2015). Interfacial n-doping using an ultrathin TiO_2_ layer for contact resistance reduction in MoS_2_. ACS Appl. Mater. Interfaces.

[11] Chee S.S., Seo D., Kim H., Jang H., Lee S., Moon S.P., Lee K.H., Kim S.W., Choi H., Ham M.H. (2019). Lowering the Schottky Barrier Height by Graphene/Ag Electrodes for High-Mobility MoS_2_ Field-Effect Transistors. Adv. Mater..

[12] Ganapathi K.L., Bhattacharjee S., Mohan S., Bhat N. (2016). High-performance HfO_2_ back gated multilayer MoS_2_ transistors. IEEE Electron. Device Lett..

[13] Das S., Chen H.Y., Penumatcha A.V., Appenzeller J. (2012). High performance multilayer MoS_2_ transistors with scandium contacts. Nano Lett..

[14] Sze S.M., Ng K.K. (2007). Physics of Semiconductor Devices.

[15] Miao J., Hu W., Jing Y., Luo W., Liao L., Pan A., Wu S., Cheng J., Chen X., Lu W. (2015). Surface plasmon-enhanced photodetection in few layer MoS_2_ phototransistors with Au nanostructure arrays. Small.

[16] Zheng J., Yan X., Lu Z., Qiu H., Xu G., Zhou X., Wang P., Pan X., Liu K., Jiao L. (2017). High-Mobility Multilayered MoS_2_ Flakes with Low Contact Resistance Grown by Chemical Vapor Deposition. Adv. Mater..

[17] Yang P., Yang A.G., Chen L. (2019). Influence of seeding promoters on the properties of CVD grown monolayer molybdenum disulfide. Nano Res..

[18] Chen J., Zhao X., Tan S.J., Xu H., Wu B., Liu B., Fu D., Fu W., Geng D., Liu Y. (2017). Chemical vapor deposition of large-size monolayer MoSe_2_ crystals on molten glass. J. Am. Chem. Soc..

[19] Liu Y., Guo J., Zhu E., Liao L., Lee S.J., Ding M., Shakir I., Gambin V., Huang Y., Duan X. (2018). Approaching the Schottky–Mott limit in van der Waals metal–semiconductor junctions. Nature.

[20] Chanana A., Mahapatra S. (2016). Prospects of zero Schottky barrier height in a graphene-inserted MoS_2_-metal interface. J. Appl. Phys..

[21] Liu T., Liu S., Tu K.H., Schmidt H., Chu L., Xiang D., Martin J., Eda G., Ross C.A., Garaj S. (2019). Crested two-dimensional transistors. Nat. Nanotechnol..

[22] Sze S.M., Coleman D.J., Loya A. (1971). Current transport in metal-semiconductor-metal (MSM) structures. Solid-State Electron..

[23] Di Bartolomeo A., Giubileo F., Luongo G., Iemmo L., Martucciello N., Niu G., Fraschke M., Skibitzki O., Schroeder T., Lupina G. (2016). Tunable Schottky barrier and high responsivity in graphene/Si-nanotip optoelectronic device. 2D Mater..

[24] Sarkar D., Xie X., Liu W., Cao W., Kang J., Gong Y., Kraemer S., Ajayan P.M., Banerjee K. (2015). A subthermionic tunnel field-effect transistor with an atomically thin channel. Nature.

[25] Shin G.H., Koo B., Park H., Woo Y., Lee J.E., Choi S.Y. (2018). Vertical-tunnel field-effect transistor based on a silicon–MoS_2_ Three-dimensional–two-dimensional heterostructure. ACS Appl. Mater. Interfaces.

[26] Wang X.F., Tian H., Liu Y., Shen S., Yan Z., Deng N., Yang Y., Ren T.L. (2019). Two-Mode MoS_2_ Filament Transistor with Extremely Low Subthreshold Swing and Record High On/Off Ratio. ACS Nano.

[27] Moriya R., Yamaguchi T., Inoue Y., Morikawa S., Sata Y., Masubuchi S., Machida T. (2014). Large current modulation in exfoliated-graphene/MoS_2_/metal vertical heterostructures. Appl. Phys. Lett..

