# Supplementary material for: A Horizontal-Gate Monolayer MoS2 Transistor Based on Image Force Barrier Reduction
Source: Nanomaterials (Basel). 2019 Sep 2;9(9):1245. doi: 10.3390/nano9091245 (PMC6780131; doi:10.3390/nano9091245)
Supplement: Supplementary file 1 [file nanomaterials-09-01245-s001.pdf]

Supplementary material

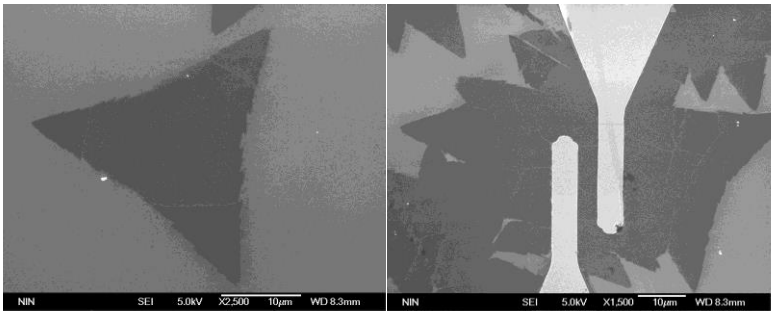

Figure S1. The SEM images of monolayer MoS_2 and transistor.

Table S1. Comparison of the performance of device with literature.

Ref.	Materials	Ion/Ioff Ratio	On-state Current(A)	Dielectric	Structure	Process complexity
Our result	MoS_2	~1×10^4	2×10^{-7}	300nm SiO_2	Horizontal-gate	I
[1]	MoS_2	~1×10^4	3×10^{-6}	90nm SiO_2	Back-gate	II
[2]	MoS_2	~1×10^4	3×10^{-7}	SiO_2	Back-gate	II
[3]	MoS_2	~10^6	1×10^{-6}	Al_2O_3/HfO_2	Dual-gate	IV
[4]	MoS_2/BP	~10^4	1×10^{-7}	Ion gel(top)	Top-gate	III
[5]	$WSe_2/SnSe_2$	~10^5	9×10^{-7}	40nm Al_2O_3 (bottom)	Back-gate	II

Process complexity progressively increases from I to IV.

Table S2. Typical values of parameters in equations.

parameters		Value
L(μm)	Length of channel	8
W(μm)	Width of channel	40
t_{ox}(nm)	Thickness of oxide	300
g_m(S)	Trans-conductance	di_{ds}/dv_{gs}
C_{ox}(F/μm^2)	Gate capacitance per unit area	1.15×10^{-4}
μ_0(cm^2/V • s)	Mobility	2.06
V_{th}(V)	Threshold voltage	-16
R_{total}(Ω)	Total resistance	v_{ds}/i_{ds}
A^* (A/(cm^2 • K^2))	Richardson's coefficient	120
k(J/K)	Boltzmann constant	1.380649×10^{-23}
T(K)	Temperature	300

References

1. Y. Vaknin, R. Dagan, and Y. Rosenwaks, Nanomaterials (Basel) **9** (6) (2019).
2. Y. H. Lee, X. Q. Zhang, W. Zhang, M. T. Chang, C. T. Lin, K. D. Chang, Y. C. Yu, J. T. Wang, C. S. Chang, L. J. Li, and T. W. Lin, Adv Mater **24** (17), 2320 (2012).
3. Pavel Bolshakov, Ava Khosravi, Peng Zhao, Paul K. Hurley, Christopher L. Hinkle, Robert M. Wallace, and Chadwin D. Young, Applied Physics Letters **112** (25) (2018).
4. Jiao Xu, Jingyuan Jia, Shen Lai, Jaehyuk Ju, and Sungjoo Lee, Applied Physics Letters **110** (3) (2017).
5. X. Yan, C. Liu, C. Li, W. Bao, S. Ding, D. W. Zhang, and P. Zhou, Small **13** (34) (2017).
